# Burden of illness in people with medically refractory epilepsy who suffer from daily to weekly seizures: 12-month follow-up of participants in the EPISODE study

**DOI:** 10.3389/fneur.2022.1012486

**Published:** 2022-10-28

**Authors:** Valérie van Hezik-Wester, Saskia de Groot, Tim Kanters, Matthijs Versteegh, Louis Wagner, Jacqueline Ardesch, Werner Brouwer, Job van Exel

**Affiliations:** ^1^Erasmus School of Health Policy & Management, Erasmus University Rotterdam, Rotterdam, Netherlands; ^2^Institute for Medical Technology Assessment, Erasmus University Rotterdam, Rotterdam, Netherlands; ^3^Kempenhaeghe and MUMC+, Academic Centre for Epileptology, Heeze, Netherlands; ^4^Stichting Epilepsie Instellingen Nederland, Zwolle, Netherlands

**Keywords:** refractory epilepsy, seizure dog, burden of illness, health-related quality of life, well-being, resource use and costs, cost-of-illness

## Abstract

**Background:**

A small group of people with epilepsy suffers from frequent seizures despite the available pharmacological and non-pharmacological interventions. The impact of epilepsy on these people extends beyond health-related quality of life (HRQoL), impacting a person's broader well-being and ability to participate in society. This study describes the burden of medically refractory epilepsy in people who suffer from daily to weekly seizures, in terms of HRQoL, well-being, and societal costs.

**Methods:**

Data from the EPISODE study on (cost-) effectiveness of seizure dogs for adults with severe medically refractory epilepsy were used, collected in 25 patients during the first 12 months before they were partnered with a certified seizure dog. Data comprised seizure diaries covering 365 days and five three-monthly surveys, including the EQ-5D-5L, QOLIE-31-P, and ICECAP-A to measure HRQoL and well-being. A societal perspective was applied to estimate costs using the iMCQ and iPCQ questionnaires about healthcare use, informal care, and productivity losses.

**Results:**

Daily seizure frequency and survey data were collected in 25 patients. A minimum of 114 observations was available for each instrument included in the survey. A total of 80% of participants experienced seizures on three or more days per week, with a median ranging from 1 to 17 seizures per seizure day. The mean EQ-5D-5L utility score was 0.682 (SD 0.235), which is considerably lower than the age-adjusted general population average. The mean QOLIE-31-P and ICECAP-A scores were 55.8 (SD 14.0) and 0.746 (SD 0.172), respectively. The average annual total cost amounted to €39,956 (range €3,804–€132,64). Informal care accounted for the largest share of costs (50%); those who received informal care reported, on average, 26 h per week (SD 30).

**Conclusions:**

Severe medically refractory epilepsy is associated with a considerable burden of illness at the patient and societal level. People with this condition have significantly reduced HRQoL and well-being and are limited in their ability to work while having substantial medical costs and a strong dependency on informal care.

## Introduction

People with medically refractory epilepsy do not achieve sustained seizure freedom despite the adequate provision of multiple pharmacological treatment regimens, known as anti-seizure medication (ASM) (1). Medically refractory epilepsy is associated with excess disability, morbidity, and mortality. It is ranked fourth in terms of disability weight among the 220 health conditions included in the Global Burden of Disease 2010 study (2). In addition to expanding opportunities for pharmacological treatment, for some people, non-pharmacological treatments such as epilepsy surgery, deep brain stimulation (DBS), vagal nerve stimulation (VNS), and the ketogenic diet can be effective alternative or complementary therapies to reduce or eliminate seizures (3, 4). Despite this, the burden of medically refractory epilepsy has remained almost unchanged for several decades.

The prevalence of medically refractory epilepsy (at least one seizure per year) is estimated to be 1.36 per 1,000 (95% CI: 1.07–1.66) in Western Europe. Of these patients, 32% had more than one seizure per week (5). This is well below the prevalence of active epilepsy (that is, newly diagnosed and medically refractory epilepsy), which is estimated at 8.23 per 1,000 (6). A study in four European countries considering all ages found that 9% of people classified with definite and probable epilepsy had daily or weekly seizures (7). While these people represent a small proportion of the epilepsy population, they account for an important share of the total burden of illness.

The health-related quality of life (HRQoL) of people with medically refractory epilepsy who suffer from frequent seizures is threatened by the seizures, the unpredictability of their occurrence, and medication side effects. Moreover, adaptations in everyday life are generally required to limit the chances of seizure-related injuries (8). Precautionary measures impact these person's mobility (e.g., restrictions on driving a car or riding a bicycle) and their ability to participate in daily activities of normal life such as sports, leisure, education, and work. Consequently, the burden of illness of people with medically refractory epilepsy who suffer from frequent seizures extends beyond HRQoL, impacting a person's broader well-being and ability to participate in society (9, 10). Therefore, the challenge for these people is balancing between staying safe and living a fulfilling life. Interventions for this patient population could be targeted at limiting the impact of seizures on everyday life and helping them maintain their independence. Examples include self-management interventions, assisted living facilities, protective gear, home safety equipment, and technical devices designed to monitor seizures and alert caregivers. More recently, there has been a growing interest in dogs trained to detect seizures and assist a person during or after a seizure.

While epilepsy is the fourth most common neurological condition, studies investigating the characteristics and burden of medically refractory epilepsy patients who suffer from frequent seizures are scarce, with most studies focusing on the pediatric population. Although previous studies have measured the HRQoL of adults with medically refractory epilepsy or the socioeconomic impact of this disease, only a few studies have assessed these issues jointly (11–14). Moreover, these studies often measured HRQoL using disease-specific rather than generic instruments, hampering the comparability of outcomes with other patient populations and their use in economic evaluations. Furthermore, well-being measures are generally not included in studies assessing the burden of illness of people with medically refractory epilepsy, while the impact of this condition on well-being is expected to be substantial. Finally, most cost-of-illness analyses in epilepsy were performed from a limited healthcare perspective and, therefore, do not account for the entire socioeconomic burden of illness (15–20). Examples of important costs not typically included are the treatment of seizure-related injuries, protective garments or home safety equipment, monitoring devices, informal care, and productivity losses in paid and unpaid work. The current study aims to describe the burden of medically refractory epilepsy in people who suffer from daily to weekly seizures in terms of HRQoL, well-being, and societal costs. By taking a societal perspective, we aim to provide a complete picture of the burden of this disease.

Data from the EPISODE study were used to study the burden of illness of people with medically refractory epilepsy who suffer from daily to weekly seizures. The EPISODE study followed 25 adults in the Netherlands with medically refractory epilepsy before and after they partnered with a certified seizure dog. People met the inclusion criteria of the EPISODE study when they had a minimum of two seizures per week despite having explored both pharmacological and non-pharmacological treatment options. Also, the seizures had to be associated with a high risk of injury or dysfunction. As such, the study population reflects a population with severe medically refractory epilepsy. Over 3 years, the study investigates the effectiveness and cost-effectiveness of seizure dogs and the effects on broader outcomes such as well-being, participation in society, and caregiver burden (21). The EPISODE study, therefore, provides unique insight into the lives of people with severe medically refractory epilepsy and the impact seizure dogs can have on their health and well-being, as well as the societal costs of this difficult-to-treat illness. While the EPISODE study is based on a relatively small sample of 25 participants, its structured set-up, longitudinal nature, as well as a broad array of instruments used offer a unique overview of the impact and costs of medically refractory epilepsy in an understudied group of patients, their environment, the health care sector, and society.

## Methods

### Data source

This study describes data from 25 participants in the EPISODE study on the (cost-) effectiveness of seizure dogs in the Netherlands who were followed over time. The main inclusion criteria for the EPISODE study were an age of 18 years or older, a confirmed diagnosis of epilepsy, an average seizure frequency of two seizures per week or more, failure of two or more ASM treatment regimens, and having had epilepsy surgery or not being eligible for epilepsy surgery (21). While there was no restriction on the type of epileptic seizures, seizures should be associated with a high risk of injury or dysfunction. The EPISODE study adopted a stepped-wedge design, wherein the order in which participants were allocated to a seizure dog was randomly assigned before the start of the study. There were no restrictions on the use of care during the study; participants received the best medical care when needed. That is, participants could receive pharmacological and non-pharmacological treatments and were allowed to use epilepsy-related technologies. Participants were followed for 3 years, during which they recorded their seizures daily using a seizure diary and completed a questionnaire every 3 months. The questionnaire included instruments for measuring seizure severity, HRQoL, well-being, healthcare use, informal care use, and productivity losses from paid and unpaid work. In addition, at baseline, sociodemographic information was collected as well as disease characteristics and details on treatment history. The rationale and design of the study are described in the study protocol (21). In the current study, the data collected during the first 12 months of the EPISODE study were used before participants were partnered with a certified seizure dog.

### Health-related quality of life

Generic outcome measures of HRQoL enable the comparison of health outcomes between different diseases and their use in economic evaluations. However, generic HRQoL measures are considered less sensitive to detecting small but clinically important health impacts related to a specific disease. Therefore, the EPISODE study included both generic and epilepsy-specific measures of HRQoL.

### EQ-5D-5L and EQ-VAS

Generic HRQoL was measured with the EQ-5D-5L questionnaire. This instrument measures HRQoL on five dimensions: mobility, self-care, usual activities, pain/discomfort, and anxiety/depression (22). Each item has five answer categories (levels): no problems, some problems, moderate problems, severe problems, and extreme problems/unable to. EQ-5D utility scores were calculated using the Dutch tariff and could take a value between −0.446 and 1 (23), with 0 representing the state of “death” and 1 representing the state of “full health”; negative values represent health states considered worse than death by the general public. In addition, overall health was assessed with the EuroQol Visual Analog Scale (EQ-VAS). EQ-VAS scores range from 0 to 100, with higher scores indicating better health. The EQ-5D-5L and EQ-VAS adopt a recall period of “today.”

#### QOLIE-31-P

Disease-specific HRQoL was assessed using a patient-weighted quality of life in epilepsy questionnaire (QOLIE-31-P). The QOLIE-31-P is designed to assess HRQoL in adults with epilepsy. Using a 100-point scale, the QOLIE-31 covers seven domains of epilepsy: seizure worry, overall quality of life, emotional well-being, energy/fatigue, cognitive functioning, medication effects, and social functioning (24). The QOLIE-31-P includes seven items asking the subjects to rate the degree of “distress” related to the topic of each domain (25). The QOLIE-31-P has a recall period of 4 weeks.

### Well-being

#### ICECAP-A

The ICEpop Capability Measure for Adults (ICECAP-A) instrument was used to measure well-being. The ICECAP-A measures capability well-being focused on the adult population and comprises five domains related to attachment, stability, achievement, enjoyment, and autonomy (26). Each of these domains has four response levels, ranging from the absence of capability to full capability. Index scores were calculated using the Dutch tariff (27). The index score was scaled to range from 0 [11111] to 1 [44444], indicating no capability and full capability, respectively. The ICECAP adopts a recall period of “at the moment.”

### Medical costs

The iMTA Medical Consumption Questionnaire (iMCQ) was used to collect data on healthcare use (28). This questionnaire includes items related to the number of visits to healthcare providers and care institutions. Furthermore, the iMCQ was applied to assess medication and home care use. The questionnaire was complemented with questions that are relevant specifically for people with epilepsy, such as consultations with a social worker or psychomotor therapist, daycare in an outpatient facility, diagnostics, procedures, and the purchase of medical equipment (e.g., protective garments, home safety equipment, monitoring devices). Healthcare use was assessed using a 3-month recall period.

Healthcare use was combined with reference prices, as provided in the Dutch costing manual, to estimate total costs (29). When reference prices were unavailable in the Dutch costing manual, prices were derived from the following sources: the Dutch Healthcare Authority (www.opendisdata.nl) for surgical procedures; the Dutch Healthcare Institute (www.gipdatabank.nl) for medical equipment, and websites from suppliers for non-medical equipment. The measurements included in this analysis were taken before participants were partnered with a certified seizure dog. When purchases were made in anticipation of the seizure dog, such as alarm systems, they were excluded from the current analysis to provide estimates of the costs of severe medically refractory epilepsy without the intervention. Drug prices were collected from the Dutch Healthcare Institute (www.farmacotherapeutischkompas.nl). To estimate drug costs without VAT, prices obtained from this website were corrected. Once every 3 months, a fee of €6.50 per drug was applied to account for pharmacy dispensing costs.

### Non-medical costs

#### Informal care

Informal care was also assessed with the iMCQ. Participants reported the cumulative number of hours of informal care received over the past 3 months, which might have been provided by more than one informal caregiver (28). In the questionnaire, informal care was defined as care falling into one of the following three categories: household activities (e.g., cleaning, grocery shopping, food preparation, taking care of children), personal care (e.g., help with dressing/undressing, washing, eating and drinking, and medication), and practical support (e.g., providing support while walking, visiting family or friends, accompanying someone to hospital appointments, managing professional help, and assisting with financial tasks). The total of informal care hours was valued using the replacement cost method (29).

#### Travel costs

Travel costs related to visits to healthcare providers were included in this analysis. Data on hospital visits, the mode of transportation, and travel distance were collected with the iMCQ. For other healthcare services, the assumption was made that participants traveled by car (and were driven by a caregiver), and the average travel distance was taken from the Dutch costing manual (29). Costs were estimated in line with the Dutch costing manual.

#### Productivity costs

Productivity costs were assessed with the iMTA Productivity Cost Questionnaire (iPCQ). The iPCQ measures absenteeism (being absent from work) and presenteeism (decreased productivity while at work), as well as losses from unpaid work (30). The questionnaire uses a recall period of 4 weeks. Presenteeism was estimated by multiplying the number of workdays during which efficiency loss was experienced by the efficiency score (0–1, with 0 representing no productivity and 1 representing total productivity), multiplied by the work h on a working day. In line with the Dutch health economic guidelines, costs of absenteeism were estimated using the friction cost method, which limits societal costs due to absenteeism to the average period required to replace an ill worker (31, 32), which was estimated to be 14.55 weeks in 2021 (33). The Dutch costing manual was followed for the monetary valuation of lost productivity (both absenteeism and presenteeism) and losses from unpaid work. As the recall of the iPCQ on short-term absenteeism and presenteeism covers 4 weeks, short-term productivity loss was extrapolated to 3 months.

Where needed, inflation correction was applied, using the general price index from the Central Bureau of Statistics of the Netherlands. All costs were expressed in 2021 euros.

### Statistical analysis

Descriptive statistics for all variables of interest were calculated. Numerical variables were shown with their mean and standard deviation, while numbers and percentages represent categorical variables.

The dataset was unbalanced due to follow-up loss, item non-response, and invalid answers. We imputed the missing values to give equal weight to all participants in the dataset. A missing value on a domain prohibited the calculation of index scores for the EQ-5D-5L and the ICECAP-A. In these cases, i.e., when partial information was available, missing domain scores were imputed using the mean of the prior and posterior observations. When the posterior observation was missing, the last observation carried forward was applied. For item non-response on the QOLIE-31-P, the scoring manual was followed. In the case of unit non-response on either of the three instruments, i.e., in the absence of information, the index score was imputed with the mean of non-missing values of the participant. The same approach was adopted to impute missing values for medical and non-medical costs, with the exception of long-term productivity loss, which was not imputed to prevent double counting as the friction cost method was applied.

Although the measurements were performed for 12 months, the data covered a maximum period of 15 months owing to the recall periods of instruments used (i.e., the questionnaire contained instruments with recall periods ranging up to 3 months). To aid interpretability and comparability, we recalculated data covering more than 12 months and presented it for 12 months where relevant. The average scores for the HRQoL and well-being measures were presented, while for resource use the accumulated yearly costs are provided. Stata/MP 16 was used to analyze the data.

## Results

### Patient population

Data were collected from 25 participants who were followed for 12 months. The mean age at baseline was 33.8 years (±12.3, range 20–57) and 56% were male. Most participants lived with their parents (48%) or partners (44%). The majority of participants did not have a paid job (76%). On average, participants have been diagnosed with epilepsy for 22.6 years (±14.1, range 2–54). Sixty-four percent was diagnosed with focal onset seizures, and generalized onset seizures were reported by 28% of participants. About one-third of participants reported daily seizures. The median 12-month seizure count was 476 (range 49–6,223), which approximates 9 seizures a week. On a seizure day, the median seizure count was three (range 1–17). Comorbidities were reported by 60% of participants, of which the majority had more than one comorbidity. The most frequently reported comorbidities were cognitive impairment (*n* = 6), developmental, learning, or behavioral disorder (*n* = 5), motor impairment (*n* = 4), respiratory disease (*n* = 4), or mental disorder (*n* = 3). Additional clinical and demographic information is provided in Table 1.

**Table 1:** Demographic characteristics at baseline (n = 25).

**Characteristics**	**N (%)**
***Sociodemographic characteristics***
**Gender**
Male	14 (56%)
Female	11 (44%)
**Age** (mean, SD, range)	33.8 (±12.3, 20–57)
**Living situation**
Alone	2 (8%)
With parents	12 (48%)
With partner and/or children	11 (44%)
**Education attainment**
Primary school or lower	4 (16%)
Secondary school	9 (36%)
Secondary vocational education	9 (36%)
Higher professional education	1 (4%)
University	2 (8%)
**Daily occupation**
Paid job	3 (12%)
Unpaid job	10 (40%)
Paid job and unpaid job	3 (12%)
None	9 (36%)
***Clinical characteristics***
**Duration of disease in years (mean, SD)**	22.6 (±14.1)
**Type of epilepsy**
Focal onset	16 (64%)
Generalized onset	7 (28%)
Unknown onset	2 (8%)
**Frequency of seizures**
Daily	8 (32%)
Three to six times a week	12 (48%)
Twice a week or less	5 (20%)
**Seizure frequency on a seizure day**^**a**^ **(median, range)**	3 (1–17)
**Comorbidity**
No comorbid conditions	10 (40%)
1 comorbid condition	2 (8%)
2–3 comorbid conditions	9 (36%)
Four or more comorbid conditions	3 (12%)
Missing	1 (4%)

a Seizures for which the participant could not record daily frequencies (i.e., because the seizures are difficult to notice or occur at a high frequency) are not considered.

### HRQoL and well-being

Table 2 shows the mean HRQoL and well-being scores reported by the 25 participants during the first year of the EPISODE study—that is, the mean across the five three-monthly measurements. For the instruments EQ-5D-5L, EQ-VAS, ICECAP-A, and QOLIE-31-P, the number of observations before imputation were 114, 116, 114, and 117 (i.e., a response rate between 91.2% and 93.6%), respectively. After imputation, a balanced dataset of 125 observations was obtained.

**Table 2:** Summary statistics for EQ-5D-5L, EQ-VAS, ICECAP-A, and QOLIE-31-P over five assessments within a 1-year follow-up (*n* = 25).

**Instrument ** ** (Possible range)**	**Average score ** ** Mean (SD)**	**Range of participant means** ** Min, Max**	**The range of individual observations Min, Max**
EQ-5D-5L (-0.446 −1)	0.682 (±0.235)	0.221, 1	−0.149, 1
EQ-VAS (0–100)	68.3 (±16.0)	33.4, 96.0	10.0, 100
ICECAP-A (0–1)	0.746 (±0.172)	0.328, 0.945	0.208, 0.964
QOLIE-31-P (0–100)	55.8 (±14.0)	29.9, 76.4	19.0, 81.9

**Figure 1:**
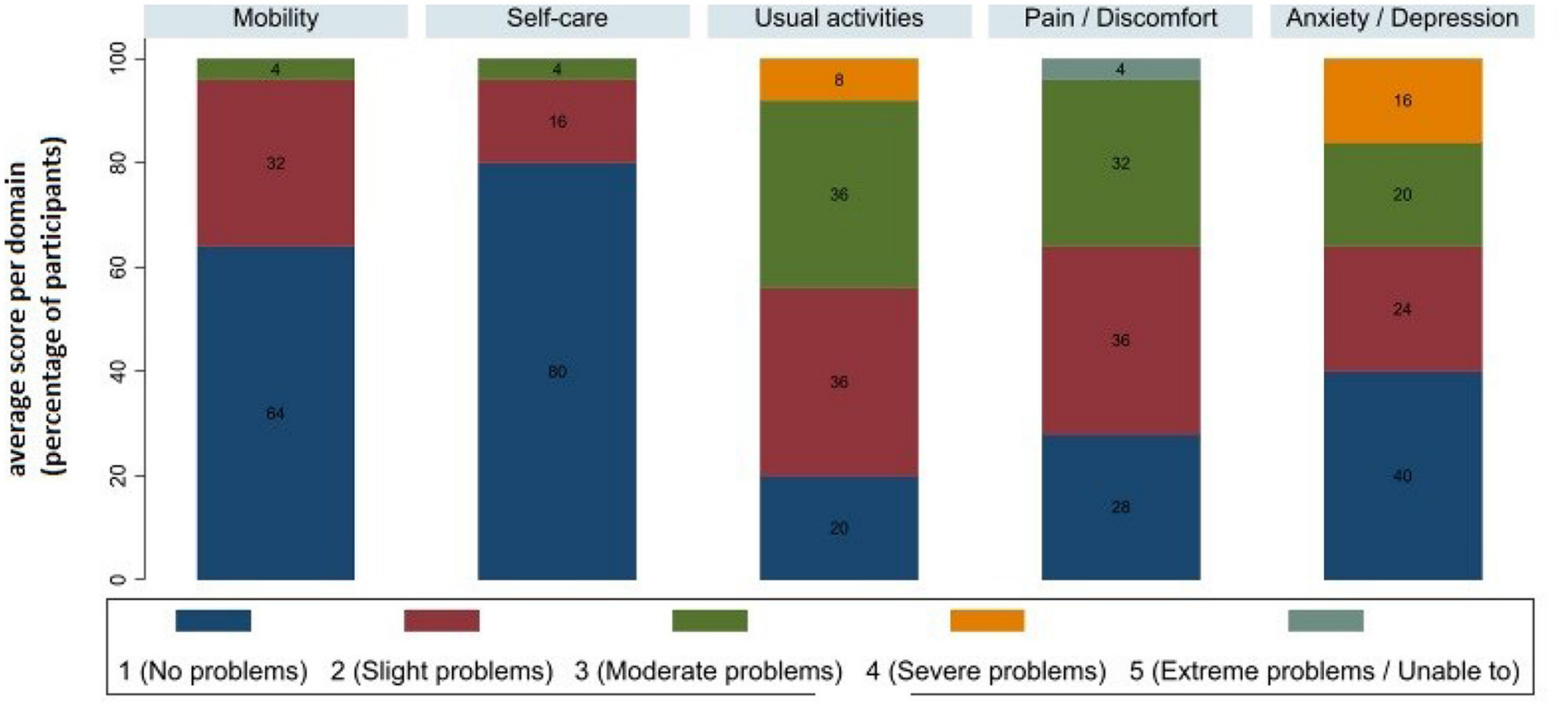
EQ-5D-5L domain scores (a higher score reflects worse health).

The mean EQ-5D-5L utility score across all observations was 0.682 (±0.235) (Table 2). Figure 1 shows the proportion of patients reporting problems by EQ−5D dimension, considering all observations. The health domain in which participants felt most impaired as usual activities, with 44% experiencing moderate or severe problems on average during the follow-up. On average, 36% of participants reported being moderately or severely anxious or depressed, and 36% reported moderate or extreme pain or discomfort. Most participants reported no problems with mobility (64%) or self-care (80%). The mean score on EQ-VAS was 68.3 (±16.0) (Table 2).

**Figure 2:**
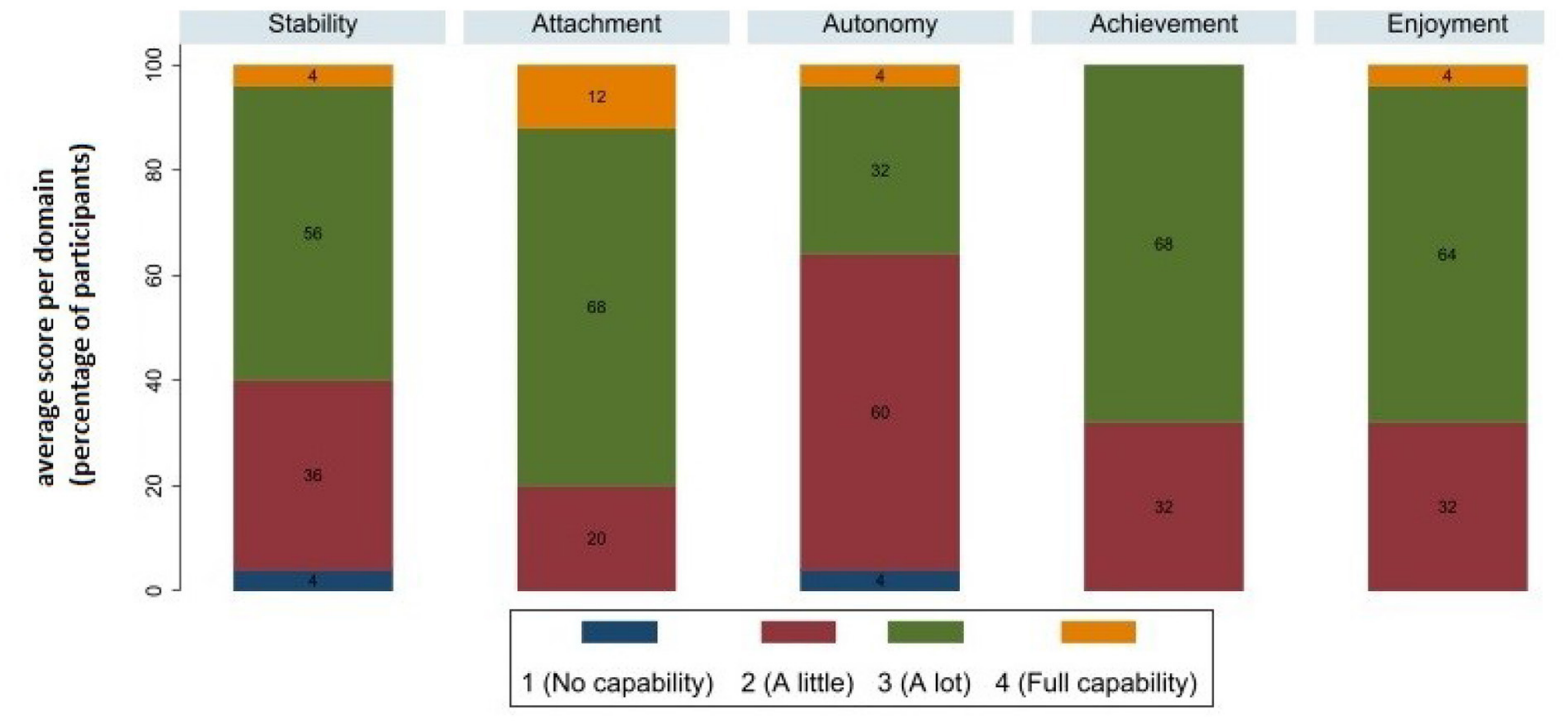
ICECAP-A domain scores (a higher score reflects better well-being).

On the ICECAP-A instrument, the average score during the first year was 0.746 (±0.172) (Table 2). Figure 2 shows the proportion of patients reporting problems by ICECAP-A dimension, considering all observations. The domains most affected were autonomy and stability, with 64% and 40% of participants reporting little or no capability on average. Attachment was the least impacted domain, with 12% of participants reporting, on average, full capability and 68% reporting many capabilities.

**Figure 3:**
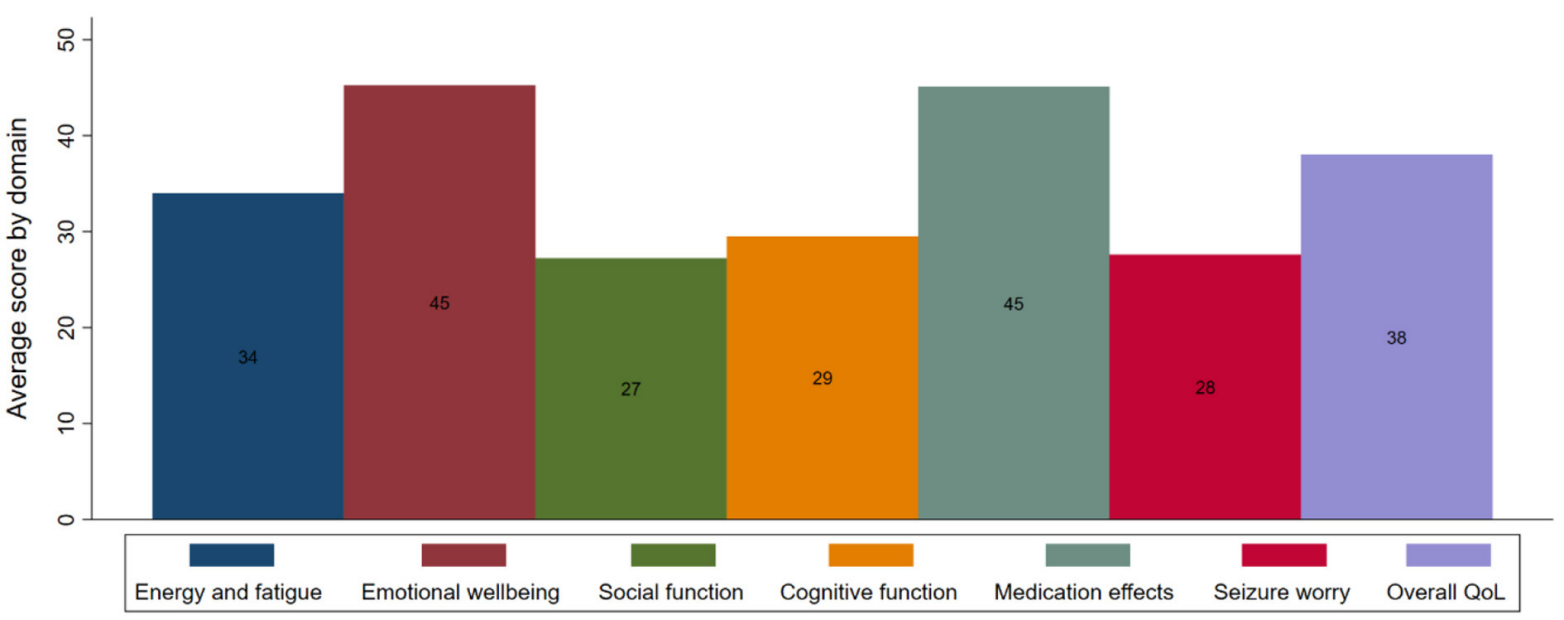
Average QOLIE-31-P domain scores (a higher score reflects a better quality of life).

The average score on the QOLIE-31-P during the first year was 55.8 (±14.0). Figure 3 shows the average domain scores on the QOLIE-31-P (before multiplication with the distress score). The lowest average scores were observed in the social function, seizure worry, and cognition domains, with scores of respectively 27 (±12), 28 (±20), and 29 (±22). Across all domains, the lowest impact was observed on emotional well-being and medication effects, both with a mean score of 45. The distress score, which reflects the weight of the degree of distress felt by the individual about each domain, revealed that participants were most distressed by cognition and seizure worry. Medication effects and emotional well-being were the least bothersome to participants.

### Medical costs

Table 3 summarizes the medical and non-medical costs in the first year of the EPISODE study. In total, annual medical costs amounted to an average of €15,823 (range of €1,617–€73,319). Overnight treatment was the most important contributor to the total medical costs (30%), but its costs varied widely among participants (range of €0– €65,627). Participants reported, on average, 25 visits to a primary care professional, mostly a physiotherapist, a general practitioner, or a psychologist, with an average of 13, 4, and 5 visits per year across the sample. Home care was used by 25% of participants, who received, on average, 3 h of assistance per week. All participants reported outpatient visits to the hospital or tertiary care center, mostly to visit a neurologist or nurse specialist (on average, nine times per year) or a social care worker (on average, four times per year). Annual costs for outpatient visits amounted to €1,412 with a range of €440–€4,683. Day treatment was used by one-third of the participants, mostly at an activity or revalidation center. Approximately half of the participants received emergency care, with ambulance calls accounting for a larger proportion of costs than emergency department visits. Diagnostic tests or medical procedures were reported by 40% of participants, most frequently electroencephalogram (EEG) diagnostics or replacement of vagal nerve stimulator (VNS) or deep brain stimulator (DBS) batteries. All participants used medication, with the average annual costs amounting to €1,837 (range €344–€4,915). 24% of participants reported medical equipment purchases, including monitoring devices, home safety equipment, and orthoses.

**Table 3:** Average resource use in natural units and costs per participant over 1 year, in euro (*n* = 25).

**Type of care**	**Unit description**	**Participants using the resource (%)**	**Average units if using**	**Range of costs ** ** if using**	**Average units per participant**	**Average costs per participant**
Primary care consultation	Appointments	100%	25 (±35)	15–6,365	25 (±35)	1,369 (±1,780)
Home care	Hours	25%	139 (±100)	220–11,511	28 (±71)	896 (±2,559)
Outpatient visit	Appointments	100%	15 (±12)	440–4,683	15 (±12)	1,412 (±1,040)
Day treatment	Days admitted	32%	50 (±55)	119–14,991	16 (±39)	1,790 (±3,971)
Overnight treatment	Nights admitted	36%	30 (±50)	423–65,627	11 (±33)	4,740 (±13,257)
Emergency care	Events	48%	7 (±9)	230–16,210	3 (±7)	1,669 (±3,529)
Medical diagnostics and interventions	Procedures	40%	1 (±1)	296–14,804	0 (±1)	1,931 (±4,760)
Medical technologies	N/A	24%	1 (±1)	132–1,200	0 (±1)	179 (±383)
Medication	N/A	100%	NA	344–4,915	NA	1,837 (±1,071)
Total medical costs				1,617–73,319		15,823 (±16,765)
Informal care	Hours	96%	1,342 (±1,561)	377–86,575	1,288 (±1,552)	20,041 (±24,131)
Productivity loss	Hours	52%	195 (±279)	121–36,952	101 (±223)	3,734 (±8,208)
Travel costs	Trips	100%	49 (±55)	5–2,785	49 (±55)	359 (±626)
Total non-medical costs				203–86,925		24,133 (±23,789)
Total costs				3,804–132,264		39,956 (±32,073)

### Non-medical costs

In total, annual non-medical costs amounted to an average of €24,133 (range €203–€86,925), comprising informal care costs, productivity losses, and travel costs. All but one participant received informal care. On average, those who received informal care reported 26 h of informal care per week (Table 3). The average annual informal care costs across the sample amounted to €20,041 (range €0–€86,575). Approximately half of the hours of informal care received comprised practical assistance. The primary caregiver was most often the participants' parent (60%), followed by the spouse or partner (40%). In total, 52% of participants experienced reduced productivity due to their health status in terms of presenteeism or absenteism in their paid or unpaid job. Two participants had stopped their paid job due to their diseases during the data collection. Overall, average annual productivity losses amounted to €3,734 with a range of €0–€36,952 per year. On average, participants made 49 trips to care providers in a year, with travel costs amounting to €359 (range €5–€2,784).

Total costs for people with severe medically refractory epilepsy amounted to €39,956 per year (range €3,804–€132,264). Non-medical costs accounted for 60% of total costs. The largest cost components were informal care (50%), inpatient care (12%), and productivity loss (9%).

## Discussion

Using data from the first year of the EPISODE study, we were able to provide a detailed account of the burden of illness of people living with severe medically refractory epilepsy. Quantifying the burden of illness can increase the awareness and understanding of the importance of (research into) interventions for this particular patient population and can be used to develop policies and inform resource allocation in this specific area. We showed that people with severe medically refractory epilepsy experience substantial deterioration in their health-related quality of life and well-being and incur considerable societal costs. With informal care accounting for 60% of total costs, people with severe medically refractory epilepsy rely heavily on their family and friends in daily life. This is reflected by the majority of participants reporting problems with autonomy (ICECAP-A) and usual activities (EQ-5D-5L).

Furthermore, while the proportion of participants with a paid job is small, productivity losses in this population should not be ignored. They can be attributed mainly to long-term absenteeism and losses from unpaid work. The disease burden, however, varied considerably between participants.

With an average EQ-5D-5L score of 0.682, participants scored considerably lower than the age-adjusted Dutch population average of 0.890 (23). Participants also scored considerably lower than other epilepsy populations. For example, a study by Wijnen et al. describes pooled data on the EQ-5D-5L and QOLIE-31-P collected in adults participating in an epilepsy self-management study in the Netherlands and the United Kingdom (34). The baseline scores were 0.86 on the EQ-5D-5L and 65.7 on the QOLIE-31-P, relative to 0.68 and 55.8 in the current study. A Dutch study looking into three types of epilepsy populations, those treated by the general practitioner, university hospital, and tertiary epilepsy center, observed the lowest quality of life scores and highest societal costs in the latter population, which most closely matches the population in the current study (11). With an average QOLIE-31 score of 62.9 and annual societal costs of €4,292 (which would compare to 5,648 in 2021 euros), their estimate of the HRQoL of people treated at an epilepsy center is higher, while their estimate of the costs is considerably lower than the findings of the current study (55.8 and €39,956) (11). These findings demonstrate the high burden of illness of people with severe medically refractory epilepsy compared to other epilepsy populations. Similarly, a study in Germany reported lower direct costs in all subgroups (35). The higher costs in our study may result from a broader approach to costing and higher disease severity.

The main strengths of this study lie in the evaluation of the burden of illness of people with severe medically refractory epilepsy from a broad societal perspective (HRQoL, well-being, and costs) and in our detailed approach to costing. The 1-year follow-up period allowed for capturing fluctuations in outcomes over time, which is relevant as seizure patterns may fluctuate, and seizure-related injuries can have a substantial yet temporal impact on HRQoL, well-being, healthcare use, reliance on informal care, and productivity.

Some limitations need to be pointed out. First, we had to base this study on a relatively small sample of 25 people with severe medically refractory epilepsy, which is an obvious limitation. People with severe medically refractory epilepsy represent a small proportion of the total epilepsy population. With this study, we were, however, able to provide insight into this understudied and hard-to-reach population. Nevertheless, it should be noted that the data used in the current study were collected in the context of the EPISODE study on seizure dogs for adults with severe medically refractory epilepsy. It is uncertain to what extent the results of the current study are generalizable to other populations with severe medically refractory epilepsy. The criteria used to determine eligibility for participating in the study extend beyond severe medically refractory epilepsy alone. For example, it was important to ensure the suitability of participants and their environment to own a seizure dog and guarantee its well-being.

Furthermore, a seizure dog may not be a desirable solution for all people with severe medically refractory epilepsy, for example, because they do not want or cannot take on the care of a seizure dog in their current living circumstances. Therefore, only a specific subset of the population eligible may have applied for participation in the EPISODE study. Such a selection bias cannot be ruled out, and the impact of these aspects on the outcomes presented remains unclear. The results might provide reliable estimates for people with frequent and severe seizures who have been exploring both pharmacological and non-pharmacological treatment options and fulfill the requirements for participating in an assistance dog program. Second, owing to the limited sample size and the probable violation of the missing at random assumption, the *ad-hoc* method of mean imputation has been applied to address missing data. This approach reduces the within-variance in the dataset, which may result in standard errors that overstate the actual precision and certainty. Third, it should be noted, that the EQ-5D-5L may not accurately reflect average HRQoL in participants as it only considers HRQoL on the day of questionnaire completion, whereas participants may have days with numerous seizures and other days where they have no seizures at all (36). A further limitation lies in the potential for double counting between the various categories of care received within hospitals or tertiary epilepsy centers. The data did not allow for linking consultations with specialists to procedures or hospitalizations. Given the considerable cost of hospitalizations and procedures relative to the cost of specialist consultation, the impact of double counting is expected to be limited. Finally, the current estimates do not help distinguish between the burden due to medically refractory epilepsy and the burden that may result from comorbid conditions. Notwithstanding these limitations, we feel this study represents an important addition to the literature on an understudied group of severely impaired people with epilepsy.

This study has investigated and detailed the burden of illness of a sample of Dutch people living with severe medically refractory epilepsy. It has been shown that the impact on these people, in terms of their health, well-being, and daily lives, as well as their caregivers, the healthcare system, and society as a whole, is substantial. Novel treatment options are needed to alleviate the burden of this disease for this patient population. If symptom relief is not possible, interventions could focus on improving coping and self-management skills and reducing the risk and severity of seizure-related injuries.

### Compliance with ethics guidelines

This article is based on previously conducted data collection and does not involve any new studies of human or animal subjects performed by any of the authors. The medical ethics committee of the Erasmus Medical Center Rotterdam has reviewed the research proposal of the EPISODE study and declared that the rules of the Medical Research Involving Human Subjects Act (also known by its Dutch abbreviation WMO) do not apply to this study. The reference number is MEC-2017-538. The medical ethics committee in Kempenhaeghe, Heeze, The Netherlands, approved the EPISODE study. The EPISODE study is registered with the Dutch Trial Register, part of the Dutch Cochrane Centre (NTR6852). Written informed consent for participation in the study is obtained from all participants before the baseline measurement.

## Data availability statement

The datasets presented in this article are not readily available because no ethical approval was obtained from respondents to share their data publicly. Requests to access the datasets should be directed to the corresponding author.

## Ethics statement

The studies involving human participants were reviewed and approved by Erasmus Medical Center Rotterdam. The patients/participants provided their written informed consent to participate in this study.

## Author contributions

VH-W, SG, TK, LW, JA, JE, WB, and MV contributed to the design of the study. VH-W was the daily coordinator of the study and drafted the manuscript. All authors commented on the manuscript and approved the final manuscript.

## Funding

The EPISODE study was funded by the Netherlands Organization for Health Research and Development (ZonMw), Grant Number 843005002. Additional funding was provided by the Innovatiefonds Zorgverzekeraars (ZN). In-kind contributions to the EPISODE study were provided by Hulphond Nederland, Bultersmekke Assistance Dogs, the Institute for Anthrozoölogy, and Erasmus School of Health Policy and Management (ESHPM).

## Conflict of interest

The authors declare that the research was conducted in the absence of any commercial or financial relationships that could be construed as a potential conflict of interest.

## Publisher's note

All claims expressed in this article are solely those of the authors and do not necessarily represent those of their affiliated organizations, or those of the publisher, the editors and the reviewers. Any product that may be evaluated in this article, or claim that may be made by its manufacturer, is not guaranteed or endorsed by the publisher.

## References

[1] Kwan P Arzimanoglou A Berg A Brodie M Allen Hauser W Mathern G. Definition of drug resistant epilepsy: consensus proposal by the *ad hoc* task force of the ILAE commission on therapeutic strategies. Epilepsia. (2010) 51:1069–77. 10.1111/j.1528-1167.2009.02397.x 19889013

[2] Salomon J Vos T Hogan DGM Naghavi M Mokdad A Begum N. Common values in assessing health outcomes from disease and injury: disability weights measurement study for the Global Burden of Disease Study 2010. The Lancet. (2012) 380:9859:2129–43. 10.1016/S0140-6736(12)61680-8 23245605 PMC10782811

[3] López González F Rodríguez Osorio X Gil-Nagel Rein A Carreño Martínez M Serratosa Fernández J Villanueva Haba V. Drug-resistant epilepsy: definition and treatment alternatives. Neurologí*a*. (2015) 30:439–46. 10.1016/j.nrleng.2014.04.002 24975343

[4] Perucca P Scheffer I Kiley M. The management of epilepsy in children and adults. Med J Aust. (2018) 208:226–33. 10.5694/mja17.00951 29540143

[5] Picot M Baldy-Moulinier M Daurès J Dujols P Crespel A. The prevalence of epilepsy and pharmacoresistant epilepsy in adults: a population-based study in a Western European country. Epilepsia. (2008) 49:1230–8. 10.1111/j.1528-1167.2008.01579.x 18363709

[6] World Health Organization. Global Campaign against Epilepsy, Programme for Neurological Diseases, Neuroscience (World Health Organization), International Bureau for Epilepsy. World Health Organization. Department of Mental Health, Substance Abuse, International Bureau of Epilepsy, International League against Epilepsy. Atlas: epilepsy care in the world. Geneva: World Health Organization (2005).

[7] Linehan C Benson A Gunko A Christensen J Sun Y Tomson T. Exploring the prevalence and profile of epilepsy across Europe using a standard retrospective chart review: challenges and opportunities. Epilepsia. (2021) 62:2651–66. 10.1111/epi.17057 34472627

[8] Loring D Meador K Lee G. Determinants of quality of life in epilepsy. Epilepsy Behav. (2004) 5:976–80. 10.1016/j.yebeh.2004.08.019 15582847

[9] Kilinç S Campbell C Guy A van Wersch A. Negotiating the boundaries of the medical model: experiences of people with epilepsy. Epilepsy Behav. (2020) 102:106674. 10.1016/j.yebeh.2019.106674 31783319

[10] McCagh J Fisk J Baker G. Epilepsy, psychosocial and cognitive functioning. Epilepsy Res. (2009) 86:1–14. 10.1016/j.eplepsyres.2009.04.007 19616921

[11] Kotsopoulos Evers S Ament A Kessels F de Krom M Twellaar M. The costs of epilepsy in three different populations of patients with epilepsy. Epilepsy Res. (2003) 54:131–40. 10.1016/S0920-1211(03)00062-7 12837564

[12] Peña P Sancho J Rufo M Martínez S Rejas J Group LSC. Driving cost factors in adult outpatients with refractory epilepsy: a daily clinical practice in clinics of neurology in Spain. Epilepsy Res. (2009) 83:133–43. 10.1016/j.eplepsyres.2008.10.004 19095410

[13] Balabanov P Zahariev Z Mateva N. Evaluation of the factors affecting the quality of life and total costs in epilepsy patients on monotherapy with carbamazepine and valproate. Folia Med. (2008) 2:18–23. 18702221

[14] Pato Pato A Cebrián Pérez E Cimas Hernando I Lorenzo González J Rodríguez Constenla I Gude Sampedro F. Analysis of direct, indirect, and intangible costs of epilepsy. Neurologia. (2011) 26:32–8. 10.1016/S2173-5808(11)70006-2 21163205

[15] Villanueva V Girón J Martín J Hernández-Pastor L Cuesta M Lahuerta J. Quality of life and economic impact of refractory epilepsy in Spain: the ESPERA study. Neurologí*a*. (2013) 28:195–204. 10.1016/j.nrleng.2012.04.013 22743210

[16] Beghi E Garattini L Ricci E Cornago D Parazzini F Group E. Direct cost of medical management of epilepsy among adults in Italy: a prospective cost-of-illness study (EPICOS). Epilepsia. (2004) 45:171–8. 10.1111/j.0013-9580.2004.14103.x 14738425

[17] Allers K Essue B Hackett M Muhunthan J Anderson C Pickles K. The economic impact of epilepsy: a systematic review. BMC Neurol. (2015) 15:245. 10.1186/s12883-015-0494-y 26607561 PMC4660784

[18] Boon P D'Havé M Van Walleghem P Michielsen G Vonck K Caemaert J. Direct medical costs of refractory epilepsy incurred by three different treatment modalities: a prospective assessment. Epilepsia. (2002) 1:96–102. 10.1046/j.1528-1157.2002.40100.x 11879393

[19] Tetto A Manzoni P Millul A Beghi E Garattini L Tartara A. The costs of epilepsy in Italy: a prospective cost-of-illness study in referral patients with disease of different severity. Epilepsy Res. (2002) 48:207–2016. 10.1016/S0920-1211(02)00013-X 11904239

[20] Guekht A Mizinova M Kaimovsky I Danilenko O Bianchi E Beghi E. The direct costs of epilepsy in Russia: a prospective cost-of-illness study from a single center in Moscow. Epilepsy Behav. (2016) 64:122–6. 10.1016/j.yebeh.2016.08.031 27736658

[21] Wester V de Groot S Kanters T Wagner L Ardesch J Corro Ramos I. Evaluating the effectiveness and cost-effectiveness of seizure dogs in people with medically refractory epilepsy in the netherlands: study protocol for a stepped wedge randomized controlled trial (EPISODE). Front Neurol. (2020) 11:3. 10.3389/fneur.2020.00003 32038471 PMC6987301

[22] Herdman M Gudex C Lloyd A Janssen M Kind P Parkin D. Development and preliminary testing of the new five-level version of EQ-5D (EQ-5D-5L). Qual Life Res. (2011) 20:1727–36. 10.1007/s11136-011-9903-x 21479777 PMC3220807

[23] Versteegh M Vermeulen K Evers S de Wit A. Dutch tariff for the five-level version of EQ-5D. Value Health. (2016) 19:343–52. 10.1016/j.jval.2016.01.003 27325326

[24] Cramer J Perrine K Devinsky O Bryant-Comstock L Meador K Hermann B. Development and cross-cultural translations of a 31-item quality of life in epilepsy inventory. Epilepsia. (1998) 39:81–8. 10.1111/j.1528-1157.1998.tb01278.x 9578017

[25] Cramer J van Hammee G Group NS. Maintenance of improvement in health-related quality of life during long-term treatment with levetiracetam. Epilepsy Behav. (2003) 4:118–23. 10.1016/S1525-5050(03)00004-0 12697135

[26] Al-Janabi H Flynn T Coast J. Development of a self-report measure of capability wellbeing for adults: the ICECAP-A. Qual Life Res. (2012) 21:167–76. 10.1007/s11136-011-9927-2 21598064 PMC3254872

[27] Rohrbach P Dingemans A Groothuis-Oudshoorn C Van Til J Essers B Van Furth E. The ICEpop capability measure for adults instrument for capabilities: development of a tariff for the dutch general population. Value Health. (2022) 25:125–32. 10.1016/j.jval.2021.07.011 35031091

[28] Bouwmans C Roijen LV Koopmanschap M Krol M Severens H Brouwer W. iMTA Medical Consumption Questionnaire (2013).

[29] Kanters T Bouwmans C van der Linden N Tan S Hakkaart-van Roijen L. Update of the Dutch manual for costing studies in health care. PLoS One. (2017) 12:e0187477. 10.1371/journal.pone.0187477 29121647 PMC5679627

[30] Bouwmans C Krol M Severens H Koopmanschap M Brouwer W Hakkaart-van Roijen L. The iMTA productivity cost questionnaire: a standardized instrument for measuring and valuing health-related productivity losses. Value Health. (2015) 18:753–8. 10.1016/j.jval.2015.05.009 26409601

[31] Zorginstituut Nederland. Guideline for conducting economic evaluations in healthcare [in Dutch: Richtlijn voor het uitvoeren van economische evaluaties in de gezondheidszorg] (2016). Available online at: https://www.zorginstituutnederland.nl/binaries/zinl/documenten/publicatie/2016/02/29/richtlijn-voor-het-uitvoeren-van-economische-evaluaties-in-de-gezondheidszorg/richtlijn-voor-het-uitvoeren-van-economische-evaluaties-in-de-gezondheidszorg.pdf (accessed October 10, 2022).27391036

[32] Koopmanschap M Rutten F Van Ineveld B Van Roijen L. The friction cost method for measuring indirect disease costs. J Health Econ. (1995) 14:171–89. 10.1016/0167-6296(94)00044-5 10154656

[33] Centraal, Bureau voor de Statistiek. Vacatures; SBI 2008; naar economische activiteit en bedrijfsgrootte. Available online at: https://opendata.cbs.nl/statline/#/CBS/nl/dataset/80473NED/table (accessed December 3, 2021).

[34] Wijnen B Mosweu I Majoie M Ridsdale L de Kinderen R Evers S. Comparison of the responsiveness of EQ-5D-5L and the QOLIE-31P and mapping of QOLIE-31P to EQ-5D-5L in epilepsy. Eur J Health Econ. (2018) 19:861–70. 10.1007/s10198-017-0928-0 28871490 PMC6008365

[35] Willems L Hochbaum M Frey K Schulz J Menzler K Langenbruch L. Multicenter, cross-sectional study of the costs of illness and cost-driving factors in adult patients with epilepsy. Epilepsia. (2022) 63:904–18. 10.1111/epi.17174 35192210

[36] Wester V de Groot S Versteegh M Kanters T Wagner L Ardesch J. Good days and bad days: measuring health-related quality of life in people with epilepsy. Value Health. (2021) 24:1470–5. 10.1016/j.jval.2021.05.001 34593170

